# Report of the Majority of the Committee on Medical Societies and Colleges on so Much of the Governor’s Message as Relates to the Cholera

**Published:** 1850-10

**Authors:** 


*Report of the Majority of the Committee on Medical Societies and Colleges on so much of the Governor's Message as relates to the Cholera.* March 25, 1850.

It will be recollected that the Governor of this State, in his annual message, recommended the Legislature to take some action to secure "the benefit of the combined experience of scientific and learned men throughout the State with respect to the origin, the cause, the progress, and the treatment of all malignant and infectious diseases."

The subject was referred by the Senate to the Committee on medical societies and colleges, of which Dr. C. D. Robinson was chairman. The majority of the Committee made a report embracing communications treating of the several points embraced in the passage of the Governor's message quoted above, by Drs. W. P. Buel, A. Vache, and Professors Reese and Clark—all of the city of New York. These papers are valuable contributions to the literature of Cholera, and the Committee, by eliciting them, have conferred a favor on the Profession and Public. Several resolutions were reported by the Committee, providing for hygienic regulations with a view to preventing the development and spread of this terrible epidemic. It is perhaps due to the adoption of effective measures to remove exciting or predisposing causes, that the cities and towns of this State have suffered so little from the disease during the past summer.

